# Exploring the Role of Low-Density Neutrophils During *Mycobacterium tuberculosis* Infection

**DOI:** 10.3389/fcimb.2022.901590

**Published:** 2022-06-21

**Authors:** Ananda N. Rankin, Skyler V. Hendrix, Sumanta K. Naik, Christina L. Stallings

**Affiliations:** Department of Molecular Microbiology, Washington University School of Medicine, Saint Louis, MO, United States

**Keywords:** neutrophil, tuberculosis, inflammation, infection, MDSC (myeloid-derived suppressor cell), low-density neutrophil

## Abstract

Tuberculosis (TB) is caused by infection with the bacterium *Mycobacterium tuberculosis* (Mtb), which primarily infects the lungs but can also cause extrapulmonary disease. Both the disease outcome and the pathology of TB are driven by the immune response mounted by the host. Infection with Mtb elicits inflammatory host responses that are necessary to control infection, but can also cause extensive tissue damage when in excess, and thus must be precisely balanced. In particular, excessive recruitment of neutrophils to the site of infection has been associated with poor control of Mtb infection, prompting investigations into the roles of neutrophils in TB disease outcomes. Recent studies have revealed that neutrophils can be divided into subpopulations that are differentially abundant in TB disease states, highlighting the potential complexities in determining the roles of neutrophils in Mtb infection. Specifically, neutrophils can be separated into normal (NDN) and low-density neutrophils (LDNs) based on their separation during density gradient centrifugation and surface marker expression. LDNs are present in higher numbers during active TB disease and increase in frequency with disease progression, although their direct contribution to TB is still unknown. In addition, the abundance of LDNs has also been associated with the severity of other lung infections, including COVID-19. In this review, we discuss recent findings regarding the roles of LDNs during lung inflammation, emphasizing their association with TB disease outcomes. This review highlights the importance of future investigations into the relationship between neutrophil diversity and TB disease severity.

## Neutrophils and Their Association With Tuberculosis

As the most abundant white blood cell in circulation, neutrophils are amongst the first cells to respond to infection and can directly control pathogen replication through phagocytosis and release of antimicrobial molecules. Neutrophils also play critical roles in initiating and maintaining inflammatory responses. Due to their inflammatory nature, when left unchecked, neutrophils can cause severe tissue damage resulting in death of the host (Ravimohan et al., 2018). Neutrophils have been identified as the most prevalent and predominantly infected cell type in the sputum, bronchoalveolar lavage (BAL) fluid, and caseum contents from resected lung tissue of active tuberculosis (TB) patients (Condos et al., 1998; Eum et al., 2010). Increased abundance of neutrophils in whole blood has also been used as a predictor for hospitalization of TB patients and is associated with poor disease outcomes (Ashitani et al., 2002; Berry et al., 2010; Ravimohan et al., 2018; Scott et al., 2020; Jones et al., 2021). Together, these studies suggest that neutrophils are associated with TB disease progression.

Several mouse strains that exhibit increased susceptibility to *Mycobacterium tuberculosis* (Mtb) infection also recruit higher numbers of neutrophils to the lungs during infection (Sugawara et al., 2003; Eruslanov et al., 2005; Keller et al., 2006; Dorhoi et al., 2010; Lázár-Molnár et al., 2010; Nandi and Behar, 2011; Niazi et al., 2015; Huynh et al., 2018; Nair et al., 2018), supporting an association between neutrophilic inflammation and poor control of Mtb infection. In some cases, depletion of neutrophils in the susceptible mice extends their survival and decreases bacterial burdens in the lungs following Mtb infection (Nandi and Behar, 2011; Dorhoi et al., 2014; Kimmey et al., 2015; Mishra et al., 2017; Nair et al., 2018; Scott et al., 2020). In addition, infection of rabbits with the hypervirulent lineage 2 Mtb strain HN878 resulted in an early increase in neutrophil recruitment and activation compared to the less virulent strain CDC1551. The early increase in neutrophil accumulation during infection with HN878 was followed by increased Mtb replication and increased pathology (Subbian et al., 2013). Furthermore, data in macaques also supports a positive correlation between increased neutrophilic infiltrate and increased disease severity (Gopal et al., 2013; Mattila et al., 2015; Gideon et al., 2019). Together, these data associate neutrophil-driven inflammation with host susceptibility to infection.

In contrast, there are some studies supporting a protective role for neutrophils during Mtb infection, specifically in blood. Neutrophils were required to restrict the growth of Mtb in human whole blood *ex vivo*, where neutrophil-depleted blood was unable to control Mtb growth compared to undepleted controls or cultures depleted of CD4^+^, CD8^+^, or CD14^+^ cells (Martineau et al., 2007; Lowe et al., 2018). Addition of viable neutrophils 96 hours post-infection to whole blood challenged with Mtb *ex vivo* significantly reduced mycobacterial growth compared to addition of necrotic neutrophils (Lowe et al., 2018). In addition, in one study, depletion of neutrophils immediately following intravenous Mtb infection in mice promoted Mtb replication in multiple organs (Pedrosa et al., 2000). These data suggest that there could be a protective role for neutrophils during Mtb infection, particularly in the context of blood infection, and may highlight differences in the roles for neutrophils during Mtb infection in the blood versus the lung. In general, the mechanisms underlying how neutrophils impact Mtb replication and disease progression remain open questions in the field.

## Discovery of Low-Density Neutrophils and Their Association With Human Disease

**Figure 1:**
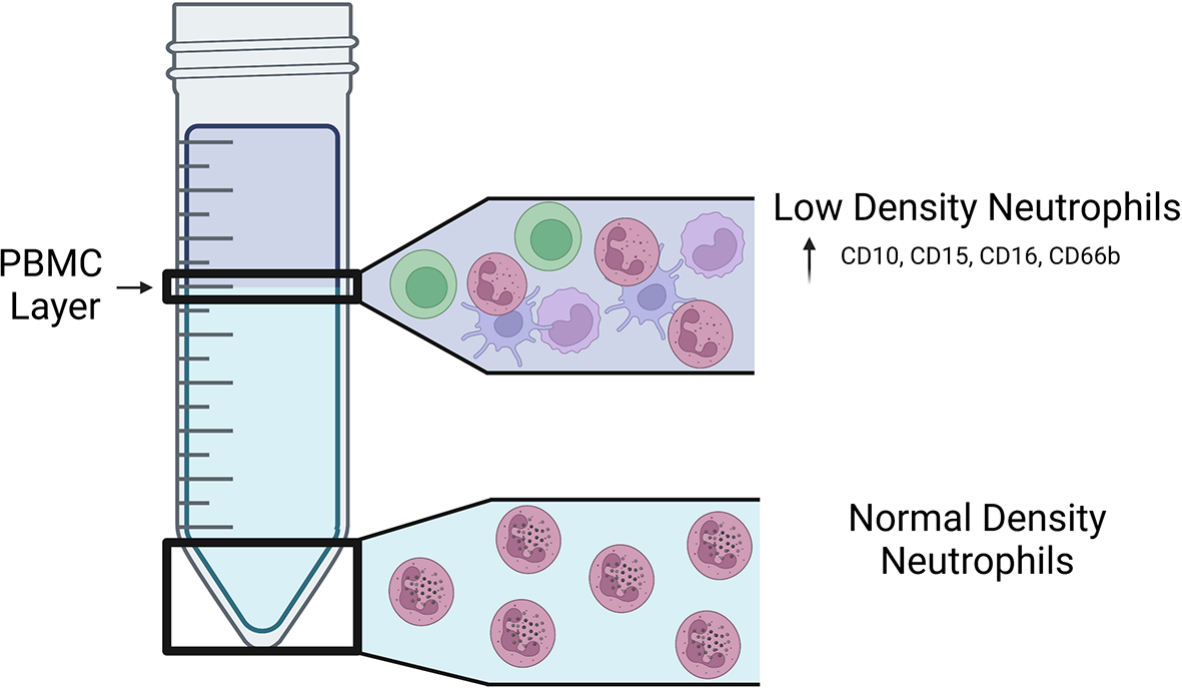
Location of LDNs and NDNs following density centrifugation. Visual representation of the fractions in which LDNs and NDNs are found on a Ficoll-Hypaque gradient. LDNs are found in the lower density PBMC layer of a Ficoll-Hypaque gradient and express increased CD10, CD15, CD16, and CD66b on their surface.

Historically, mature human neutrophils have been considered a homogenous cell population that are defined by multilobed-nuclei, intracellular granules, and cell surface expression of CD15, CD16, CD45, CD11b, CD66b, and CD10 (Fortunati et al., 2009; Marini et al., 2017; Negorev et al., 2018). Studies in recent years, however, have revealed that there are several subsets of mature neutrophils that exist in humans, and this neutrophil heterogeneity is associated with inflammation and the outcomes of pulmonary and extrapulmonary diseases. Early human neutrophil studies relied on Ficoll-Hypaque density centrifugation as a method of separating red blood cells from immune cells in the blood (Böyum, 1968; Ferrante and Thong, 1980). Peripheral blood mononuclear cells (PBMCs) and polymorphonuclear cells (PMNs), including neutrophils and other granulocytes, sediment in distinct layers based on the respective cell type’s density, where PMNs have greater density than PBMCs (Ferrante and Thong, 1980) (**Figure 1**). In 1986, Hacbarth and Kajdacsy-Balla were the first to describe neutrophils with “lower buoyant density” contaminating the PBMC cell density layer in Ficoll-Hypaque preparations of blood from patients with systemic lupus erythematosus (SLE), rheumatoid arthritis (RA), and acute rheumatic fever (ARF) (Hacbarth and Kajdacsy-Balla, 1986). This subset of neutrophils became known as low-density neutrophils (LDNs), often used interchangeably with low-density granulocytes (LDGs), to distinguish them from the classical normal-density neutrophils (NDNs) (Hacbarth and Kajdacsy-Balla, 1986; Sun, 2022).

Similar to NDNs, mature LDNs have multilobed-nuclei, intracellular granules, and surface expression of CD45, CD11b, CD15, CD16, CD66b and CD10 (Kishimoto et al., 1989; Nakayama et al., 2001; van Lochem et al., 2004; Schmidt et al., 2012). Mature LDNs express higher levels of CD15 and CD16b on their surface compared to NDNs, as well as CD66b, which is also a marker for activated NDNs (Deng et al., 2016; La Manna et al., 2019; Blanco-Camarillo et al., 2021; Hardisty et al., 2021; Reyes et al., 2021). When healthy individuals were administered granulocyte colony-stimulating factor-treated (G-CSF) for five days before isolating LDNs from the peripheral blood, both mature (CD10^+^) and immature (CD10^-^) populations of LDNs were identified (Marini et al., 2017). In addition, recent studies identified immature LDNs in the blood of patients with acute respiratory distress syndrome (ARDS) with and without comorbid COVID-19 (Reyes et al., 2021). Other surface markers used to distinguish LDNs from NDNs vary based on the study and disease state (Hardisty et al., 2021); however, there are currently no distinct surface markers that unequivocally distinguish LDNs from NDNs.

LDNs account for approximately 5% of cells isolated from the PBMC cell density layer in healthy individuals (Hacbarth and Kajdacsy-Balla, 1986; Blanco-Camarillo et al., 2021; Hardisty et al., 2021). The number of LDNs in the blood increases when homeostasis is disrupted, such as during lung infection with Mtb or SARS-CoV-2, lung cancer, diabetes, and in patients with autoimmune diseases (Hacbarth and Kajdacsy-Balla, 1986; Cohen et al., 2019; Reyes et al., 2021; Valadez-Cosmes et al., 2021). Determining the exact source of LDNs has been difficult due to their lack of distinct surface markers and fluctuating levels in circulation between healthy individuals and patients. Incubating NDNs with serum from patients with SLE or severe fever with thrombocytopenia syndrome (SFTS), a disease caused as a result of infection with the SFTS bunyavirus, decreases the buoyant density of NDNs, indicating that NDNs could be a source of LDNs and an extracellular factor is sufficient to induce a physiological change in neutrophils that results in a lower density (Hacbarth and Kajdacsy-Balla, 1986; Li et al., 2019). However, whether the transition from NDN to LDN occurs *in vivo*, if LDNs can be converted to NDNs, or if LDNs and NDNs can originate from the same neutrophil progenitors has yet to be determined. It is still unclear how inflammatory responses result in increased accumulation of LDNs in the blood and whether changes occur in the bone marrow microenvironment to produce LDNs. Compounding the complexity of determining the source of LDNs, NDNs in human blood samples have been shown to spontaneously shift densities in response to delayed sample processing, which must be taken into consideration when conducting studies on LDNs (McKenna et al., 2009).

## LDN Characteristics and Responses

Since their discovery, many groups have worked to determine the function of LDNs in the body. In a recent study, Blanco-Camarillo et al. compared LDNs and NDNs from the blood of healthy individuals and found that LDNs exhibited higher levels of phagocytosis and reactive oxygen species (ROS) production, but similar production of neutrophil extracellular traps (NETs) compared to NDNs. The findings of this study, however, conflicted with a study published by Hassani et al., in which they compared the 20% highest and lowest density neutrophils from healthy individuals and found that there was no difference in phagocytosis (Hassani et al., 2020). Mature LDNs from healthy individuals have also been shown to suppress lymphocyte proliferation (Marini et al., 2017; Hassani et al., 2020; Seman et al., 2021), indicating that in some contexts LDNs may act in an anti-inflammatory fashion. PMN myeloid-derived suppressor cells (PMN-MDSCs) that inhibit T cell proliferation have also been isolated from the low-density Ficoll-Hypaque layer and express CD11b, CD15, and CD66b on the surface (Schmielau and Finn, 2001; Bronte et al., 2016; Seman and Robinson, 2021). Whether LDNs and PMN-MDSCs are distinct cell populations that migrate with PBMCs in the Ficoll-Hypaque gradient is still unknown, but if this is the case, different studies may isolate different proportions of these cell types, which would affect functional analyses.

LDN responses have also been studied in the context of autoimmune diseases and infection. LDNs isolated from patients with SLE, RA, or SFTS exhibited increased production of proinflammatory cytokines, type 1 interferons, phagocytic activity, and release of NETs compared to NDNs (Denny et al., 2010; Lood et al., 2016; Midgley and Beresford, 2016; Li et al., 2019). The NETs released from LDNs during *Escherichia coli* infection were found to suppress the antibacterial activity of monocytes in a co-culture system; however, it is not known whether this differs from NETs released by NDNs (Seman et al., 2021). LDNs isolated from patients with solid tumor cancers, HIV, or graft-versus-host disease (GvHD) suppressed T cell proliferation and function (Schmielau and Finn, 2001; Cloke et al., 2012; Brandau et al., 2013; Cloke et al., 2013; Rieber et al., 2014; Farzeen, 2016; Scapini et al., 2016; Silvestre-Roig et al., 2019). Mature LDNs from the blood of patients with ARDS with and without comorbid COVID-19 expressed higher levels of the platelet activation marker CD41 than immature LDNs, formed aggregates with platelets, and activated prothrombotic pathways in COVID-19 patients (Reyes et al., 2021). Together these data demonstrate that LDNs can impact multiple arms of the immune system, possibly in a context-dependent manner.

## LDNs in TB

LDN numbers are significantly elevated in the peripheral blood of active TB patients who have not received anti-tuberculous therapy compared to healthy controls (Deng et al., 2016; La Manna et al., 2019). In addition, the number of LDNs per one thousand neutrophils is significantly elevated in active TB patients compared to healthy controls, demonstrating that even in the context of increased total neutrophils in the blood of active TB patients, the proportion of LDNs was also elevated (Deng et al., 2016). Comparison of moderate and advanced cases of active TB revealed that patients with advanced disease had significantly more LDNs than those with moderate disease (Deng et al., 2016). Furthermore, comparing LDNs in patients at different stages of anti-TB treatment revealed that the levels of LDNs decreased over the course of treatment, supporting the notion that increased levels of LDNs are associated with worsening TB disease severity (Deng et al., 2016). Based on flow cytometric analyses of markers associated with activation (CD66b and CD62L shedding), immaturity (CD33), and maturity (CD15 and CD16), LDNs from active TB patients expressed higher levels of CD15, CD33, CD66b, CD16, and lower levels of CD62L compared to autologous NDNs (Deng et al., 2016; La Manna et al., 2019). These results suggest that LDNs from active TB patients are more activated than NDNs and likely contain a mixed population of both mature and immature cells, consistent with what has been reported for LDNs in other diseases (Hassani et al., 2020; Blanco-Camarillo et al., 2021; Cabrera et al., 2021).

## Induction of LDNs During Mtb Infection *in vitro*

**Figure 2:**
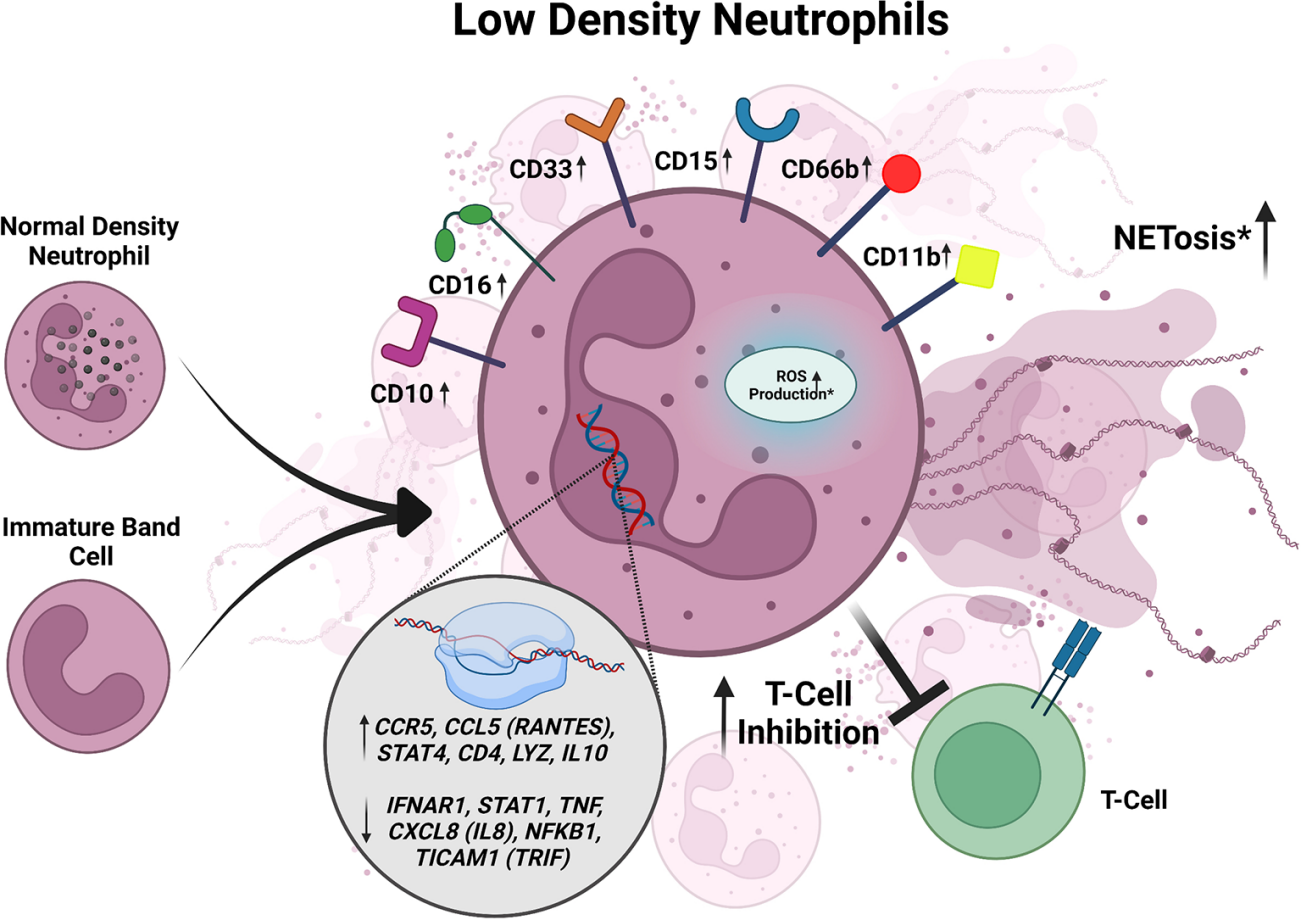
Summary of characteristics of LDNs described in active TB. LDNs may be derived from NDNs or an unknown neutrophil progenitor. In active TB, LDNs express increased CD10, CD11b, CD15, CD16, CD33, and CD66b. When exposed to Mtb *in vitro*, LDNs exhibit inhibition of T cell responses. LDNs have also been shown to exhibit higher levels of NETosis and ROS production in response to Mtb infection when compared to NDNs, but the data for these responses is inconsistent between studies and requires further investigation. LDNs also have increased expression of *CCL5*, *STAT4*, *CD4*, *LYZ*, *IL-10*, *IFNAR1*, *STAT1*, *CCL5 (RANTES)*, *STAT4*, *TNF*, and *CXCL8*. Conflicting data is designated by *.

*In vitro* Mtb infection of healthy donor peripheral blood samples resulted in a significant increase in the LDN population in both a time- and dose-dependent manner (Deng et al., 2016), suggesting that Mtb infection induces the formation of LDNs (**Figure 2**). Treatment with the ROS scavenger N-acetyl-L-cysteine or inhibition of NADPH oxidase inhibits LDN formation in Mtb-infected peripheral blood samples (Su et al., 2019). ROS activates NETosis and inhibition of NETosis, without affecting ROS levels, *via* the protein arginine deiminase (PAD) inhibitor Cl-amidine prevented LDN formation in Mtb infected peripheral blood *in vitro* (Su et al., 2019). This work suggests a possible mechanism for LDN induction whereby Mtb infection of blood cells induces ROS production, leading to NETosis and subsequent induction of LDN development. The role of NETs in the formation of LDNs is particularly intriguing given the recent association of NETs with lesions in active TB patients not responding to antibiotic therapy (Moreira-Teixeira et al., 2020).

## Activity of LDNs Isolated From TB Patients

To determine if LDNs could have functional consequences on TB disease outcomes, their responses during *Mtb* infection have been monitored in multiple studies, but with contradicting results (**Figure 2**). In the first *in vitro* studies assessing Mtb uptake, LDNs from TB patients were associated with more bacteria than autologous NDNs, as measured by flow cytometry, although this did not affect bacterial clearance (Deng et al., 2016). In contrast, a different study found there was reduced phagocytosis of Mtb by LDNs compared to NDNs using confocal microscopy (La Manna et al., 2019). Intracellular ROS, as measured by flow cytometry, was significantly increased in LDNs isolated from the peripheral blood of active TB patients compared to autologous NDNs and NDNs from healthy donors (Deng et al., 2016). However, another group reported no intracellular ROS production in LDNs from TB patients, with and without phorbol-myristate acetate (PMA) stimulation, using a redox-sensitive dye and flow cytometry (La Manna et al., 2019). Spontaneous NETosis, assessed by quantifying myeloperoxidase (MPO) and DAPI double positive structures, was significantly increased in LDNs isolated from active TB patients compared to NDNs, whereas upon stimulation with PMA, NETosis by LDNs was significantly decreased compared to NDNs (Su et al., 2019). However, using flow cytometry another group found that spontaneous and stimulated NETosis of LDNs from TB patients was reduced compared to autologous NDNs (La Manna et al., 2019). All of the published studies on LDNs from active TB patients identified LDNs based on CD15 positivity and lower density buoyancy, but used varying multiplicities of infection, lengths of infection time, methods to stimulate the neutrophils before measuring NETosis, and ROS-sensitive fluorescent dyes (Deng et al., 2016; La Manna et al., 2019; Su et al., 2019), which may contribute to the differences in results.

Of 84 total cytokine and chemokine transcripts analyzed by qRT-PCR in active TB patients, there were twelve differentially expressed genes between LDNs and NDNs (La Manna et al., 2019). *CCL5* (*RANTES*), *CCR5, CD4*, *STAT4*, *IL10*, and *LYZ* were significantly upregulated in LDNs, whereas *CXCL8* (*IL8*), *STAT1*, *IFNAR1*, *NFKB1*, *TICAM1*, and *TNF* were significantly downregulated (**Figure 2**) (La Manna et al., 2019). *CCL5*, *STAT4*, *CD4*, *LYZ* upregulation in neutrophils have been associated with inflammation and neutrophil antimicrobial activity (Flanagan and Lionetti, 1955; Pan et al., 2000; Hartl et al., 2008; Keeter et al., 2020; Mehrpouya-Bahrami et al., 2021). IL-10 and IFNAR1 signaling have been associated with susceptibility to TB and poor outcomes of disease (Verbon et al., 1999; Bonecini-Almeida et al., 2004; Jamil et al., 2007; Dorhoi et al., 2014; Huynh et al., 2018; Zhang et al., 2018; Moreira-Teixeira et al., 2020). STAT1 signaling is necessary for the control of Mtb in mice (Sugawara et al., 2004) and polymorphisms in STAT1 have been associated with poor control of mycobacterial diseases (Dupuis et al., 2001; Chapgier et al., 2006). CCL5 (RANTES), STAT4, and TNF are generally associated with better control of TB (Flynn et al., 1995; Gómez-Reino et al., 2003; Sugawara et al., 2004; Askling et al., 2005; Stegelmann et al., 2005; Lee et al., 2008; Vesosky et al., 2010). In some reports, CXCL8 (IL8) has been shown to be important for TB control (O’Kane et al., 2007; Krupa et al., 2015; Larsen et al., 1995), but is also found to be upregulated in granulomas containing excessive neutrophils (Bergeron et al., 1997; Hashemian et al., 2014). It is unknown whether the differential expression of CCL5 (RANTES), CCR5, CD4, STAT4, IL10, LYZ, CXCL8 (IL8), STAT1, IFNAR1, NFKB1, TICAM1, and TNF between LDNs and NDNs is functionally relevant during Mtb infection, but these data open potential areas of inquiry into how LDNs could be impacting immune responses to Mtb.

When PBMCs isolated from patients with active TB were infected with *Mycobacterium bovis* Bacillus Calmette–Guérin (BCG) in the presence of LDNs or NDNs *in vitro*, T cell proliferation was lower in the presence of LDNs compared to NDNs (**Figure 2**) (La Manna et al., 2019). Although *in vitro* work showed that LDNs upregulated IL-10 expression, addition of an anti-IL-10 antibody did not change the differences in the T cell proliferation observed in the presence of LDNs versus NDNs (La Manna et al., 2019), indicating that the effect of LDNs on T cell proliferation in this assay is IL-10-independent. The association between LDNs and T cell responses during Mtb infection has also been analyzed in active TB patients using the T-SPOT.TB test to assess IFN-γ secretion by T cells in response to the Mtb antigens ESAT-6 and CFP-10 (Rao et al., 2021). Active TB patients with high LDN frequencies had lower numbers of IFN-ү-secreting cells than patients with low LDN frequencies (**Figure 2**) (Rao et al., 2021). Removal of LDNs from the peripheral blood samples increased the number of IFN-ү secreting cells in the samples (Rao et al., 2021), indicating that LDNs were negatively correlated with T cell responses required to control Mtb infection. The LDNs from active TB patients expressed higher levels of PD-L1, which inhibits T cell proliferation, on their surface compared to NDNs from healthy controls, suggesting a model in which LDNs could prevent IFN-γ secretion by inhibiting T cell expansion *via* PD-L1 (Rao et al., 2021). Antibody-mediated inhibition of PD-L1 signaling recapitulated the removal of LDNs from the PBMC samples from active TB patients with high LDN frequencies (Rao et al., 2021). Similarly, addition of LDNs to PBMC samples from TB patients with low LDN frequencies decreased IFN-ү secreting cell numbers in response to Mtb antigen and addition of anti-PD-L1 antibody rescued IFN-ү secreting cell numbers to that of the control (Rao et al., 2021). If LDNs contribute to T cell suppression *in vivo*, this could explain one reason the percentage of LDNs in the blood correlates with the increased severity of TB disease. Notably, PMN-MDSCs also express PD-L1 on their surface and have been identified in active TB patients (McNab et al., 2011; du Plessis et al., 2013; El Daker et al., 2015). Further studies would be required to determine whether PMN-MDSCs co-isolated with LDNs contribute to the observed effects on T cells.

## Discussion

LDNs have been identified and characterized in several diseases; however, there are still many remaining questions regarding their development, maintenance, and roles in immunity. Thus far, LDNs have only been analyzed in the blood due to limitations inherent to the way LDNs are distinguished from NDNs, based on lower density and migration in the PBMC layer on a Ficoll-Hypaque gradient. Therefore, it is still unknown whether LDNs are present in tissues. During active TB, granulocytes in the blood are recruited to the lungs, but whether this occurs for LDNs and how their properties would change once in the tissue remains an open question. Further work is needed to determine ways to identify and isolate LDNs from tissue, possibly by positively selecting for granulocytes followed by performing density centrifugation.

In general, very little is known about LDNs in TB, and the data that has been published varies between groups. One limitation to mechanistic studies is the absence of identification of an analogous population in small animal models of TB. LDNs have been described in mouse models of *S. aureus*-induced sepsis, which may serve as a foundation for studying LDNs in mouse models of TB (Cohen et al., 2019; Takizawa et al., 2021). Although LDNs have not specifically been identified in mouse models of Mtb infection, neutrophil diversity has been observed in mice during infection. Multiple studies have described the accumulation of a Ly6G^Mid^ population of neutrophils in susceptible mouse models of TB, where increased abundance of Ly6G^Mid^ neutrophils was positively correlated with higher Mtb burden in the lungs (Obregón-Henao et al., 2013; Tsiganov et al., 2014; Lovewell et al., 2021). Tsiganov et al. found that these Ly6G^Mid^ cells were heterogenous in nuclear structure, had significantly lower side scatter than Ly6G^high^ neutrophils, indicating lower cell granularity, and expressed both neutrophil and monocyte markers (Tsiganov et al., 2014). Further work will be needed to determine whether Ly6G^Mid^ neutrophils in mice are analogous to LDNs in humans or whether they represent yet another example of neutrophil diversity. In addition to LDNs and PMN-MDSCs, other subsets of neutrophils have been identified in humans during cancer, anti-neutrophil cytoplasmic antibody-associated systemic vasculitis, and bacterial infections with *Staphylococcus aureus* and *E. coli*, including N1/N2 neutrophils, C177 expressing neutrophils, and Olfactomedin-4 expressing neutrophils (Rarok et al., 2002; Fridlender et al., 2009; Hu et al., 2009; Liu et al., 2012; Liu et al., 2013; Grieshaber-Bouyer and Nigrovic, 2019; Filep and Ariel, 2020). However, whether these neutrophil subpopulations are present during Mtb infection has yet to be determined. Given that neutrophils are the predominant immune cell type present and infected in the lungs of active TB patients, understanding neutrophil diversity and the roles for various neutrophil subpopulations during Mtb infection will be important to dissect the immune responses that either control or promote Mtb pathogenesis.

## Author Contributions

All authors participated in the conceptualization and writing of this mini-review. All authors contributed to the article and approved the submitted version.

## Funding

CS is supported by NIHgrants AI132653, AI132697, and AI142784, as well as a Burroughs Wellcome Fund Investigators in the Pathogenesis of Infectious Disease Award. SH was supported by NIH grant GM007067. AR is supported by NSF GRF DGE-2139839 and DGE-1745038. SN is supported by a Stephen I. Morse Postdoctoral Fellowship.

## Conflict of Interest

The authors declare that the research was conducted in the absence of any commercial or financial relationships that could be construed as a potential conflict of interest.

## Publisher’s Note

All claims expressed in this article are solely those of the authors and do not necessarily represent those of their affiliated organizations, or those of the publisher, the editors and the reviewers. Any product that may be evaluated in this article, or claim that may be made by its manufacturer, is not guaranteed or endorsed by the publisher.

## References

[B1] Ashitani J. Mukae H. Hiratsuka T. Nakazato M. Kumamoto K. Matsukura S. (2002). Elevated Levels of α-Defensins in Plasma and BAL Fluid of Patients With Active Pulmonary Tuberculosis. Chest 121, 519–526. doi: 10.1378/chest.121.2.519 11834667

[B2] Askling J. Fored C. M. Brandt L. Baecklund E. Bertilsson L. Cöster L.. (2005). Risk and Case Characteristics of Tuberculosis in Rheumatoid Arthritis Associated With Tumor Necrosis Factor Antagonists in Sweden. Arthritis Rheum. 52, 1986–1992. doi: 10.1002/art.21137 15986370

[B3] Bergeron A. Bonay M. Kambouchner M. Lecossier D. Riquet M. Soler P.. (1997). Cytokine Patterns in Tuberculous and Sarcoid Granulomas: Correlations With Histopathologic Features of the Granulomatous Response. J. Immunol. 159, 3034–3043. 9300729

[B4] Berry M. P. R. Graham C. M. McNab F. W. Xu Z. Bloch S. A. A. Oni T.. (2010). An Interferon-Inducible Neutrophil-Driven Blood Transcriptional Signature in Human Tuberculosis. Nature 466, 973–977. doi: 10.1038/nature09247 20725040 PMC3492754

[B5] Blanco-Camarillo C. Alemán O. R. Rosales C. (2021). Low-Density Neutrophils in Healthy Individuals Display a Mature Primed Phenotype. Front. Immunol. 12. doi: 10.3389/fimmu.2021.672520 PMC8285102 34276661

[B6] Bonecini-Almeida M. G. Ho J. L. Boéchat N. Huard R. C. Chitale S. Doo H.. (2004). Down-Modulation of Lung Immune Responses by Interleukin-10 and Transforming Growth Factor Beta (TGF-Beta) and Analysis of TGF-Beta Receptors I and II in Active Tuberculosis. Infect. Immun. 72, 2628–2634. doi: 10.1128/IAI.72.5.2628-2634.2004 15102771 PMC387880

[B7] Böyum A. (1968). Separation of Leukocytes From Blood and Bone Marrow. Introduction. Scand. J. Clin. Lab. Invest. Suppl. 97, 7. 5707208

[B8] Brandau S. Dumitru C. A. Lang S. (2013). Protumor and Antitumor Functions of Neutrophil Granulocytes. Semin. Immunopathol. 35, 163–176. doi: 10.1007/s00281-012-0344-6 23007469

[B9] Bronte V. Brandau S. Chen S.-H. Colombo M. P. Frey A. B. Greten T. F.. (2016). Recommendations for Myeloid-Derived Suppressor Cell Nomenclature and Characterization Standards. Nat. Commun. 7, 12150. doi: 10.1038/ncomms12150 27381735 PMC4935811

[B10] Cabrera L. E. Pekkarinen P. T. Alander M. Nowlan K. H. A. Nguyen N. A. Jokiranta S.. (2021). Characterization of Low-Density Granulocytes in COVID-19. PLoS Pathog. 17, e1009721. doi: 10.1371/journal.ppat.1009721 34228753 PMC8284631

[B11] Chapgier A. Boisson-Dupuis S. Jouanguy E. Vogt G. Feinberg J. Prochnicka-Chalufour A.. (2006). Novel STAT1 Alleles in Otherwise Healthy Patients With Mycobacterial Disease. PLoS Genet. 2, e131. doi: 10.1371/journal.pgen.0020131 16934001 PMC1550284

[B12] Cloke T. Munder M. Bergin P. Herath S. Modolell M. Taylor G.. (2013). Phenotypic Alteration of Neutrophils in the Blood of HIV Seropositive Patients. PLoS One 8, e72034. doi: 10.1371/journal.pone.0072034 24039734 PMC3767740

[B13] Cloke T. Munder M. Taylor G. Müller I. Kropf P. (2012). Characterization of a Novel Population of Low-Density Granulocytes Associated With Disease Severity in HIV-1 Infection. PLoS One 7, e48939. doi: 10.1371/journal.pone.0048939 23152825 PMC3496742

[B14] Cohen T. S. Takahashi V. Bonnell J. Tovchigrechko A. Chaerkady R. Yu W.. (2019). Staphylococcus Aureus Drives Expansion of Low-Density Neutrophils in Diabetic Mice. J. Clin. Invest. 129, 2133–2144. doi: 10.1172/JCI126938 30985291 PMC6486344

[B15] Condos R. Rom W. N. Liu Y. M. Schluger N. W. (1998). Local Immune Responses Correlate With Presentation and Outcome in Tuberculosis. Am. J. Respir. Crit. Care Med. 157, 729–735. doi: 10.1164/ajrccm.157.3.9705044 9517583

[B16] Deng Y. Ye J. Luo Q. Huang Z. Peng Y. Xiong G.. (2016). Low-Density Granulocytes Are Elevated in Mycobacterial Infection and Associated With the Severity of Tuberculosis. PLoS One 11, e0153567. doi: 10.1371/journal.pone.0153567 27073889 PMC4830625

[B17] Denny M. F. Yalavarthi S. Zhao W. Thacker S. G. Anderson M. Sandy A. R.. (2010). A Distinct Subset of Proinflammatory Neutrophils Isolated From Patients With Systemic Lupus Erythematosus Induces Vascular Damage and Synthesizes Type I IFNs. J. Immunol. 184, 3284–3297. doi: 10.4049/jimmunol.0902199 20164424 PMC2929645

[B18] Dorhoi A. Desel C. Yeremeev V. Pradl L. Brinkmann V. Mollenkopf H.-J.. (2010). The Adaptor Molecule CARD9 Is Essential for Tuberculosis Control. J. Exp. Med. 207, 777–792. doi: 10.1084/jem.20090067 20351059 PMC2856020

[B19] Dorhoi A. Yeremeev V. Nouailles G. Weiner J. Jörg S. Heinemann E.. (2014). Type I IFN Signaling Triggers Immunopathology in Tuberculosis-Susceptible Mice by Modulating Lung Phagocyte Dynamics. Eur. J. Immunol. 44, 2380. doi: 10.1002/eji.201344219 24782112 PMC4298793

[B20] du Plessis N. Loebenberg L. Kriel M. von Groote-Bidlingmaier F. Ribechini E. Loxton A. G.. (2013). Increased Frequency of Myeloid-Derived Suppressor Cells During Active Tuberculosis and After Recent Mycobacterium Tuberculosis Infection Suppresses T-Cell Function. Am. J. Respir. Crit. Care Med. 188, 724–732. doi: 10.1164/rccm.201302-0249OC 23885784

[B21] Dupuis S. Dargemont C. Fieschi C. Thomassin N. Rosenzweig S. Harris J.. (2001). Impairment of Mycobacterial But Not Viral Immunity by a Germline Human STAT1 Mutation. Science 293, 300–303. doi: 10.1126/science.1061154 11452125

[B22] El Daker S. Sacchi A. Tempestilli M. Carducci C. Goletti D. Vanini V.. (2015). Granulocytic Myeloid Derived Suppressor Cells Expansion During Active Pulmonary Tuberculosis Is Associated With High Nitric Oxide Plasma Level. PloS One 10, e0123772. doi: 10.1371/journal.pone.0123772 25879532 PMC4400140

[B23] Eruslanov E. B. Lyadova I. V. Kondratieva T. K. Majorov K. B. Scheglov I. V. Orlova M. O.. (2005). Neutrophil Responses to Mycobacterium Tuberculosis Infection in Genetically Susceptible and Resistant Mice. Infect. Immun. 73, 1744–1753. doi: 10.1128/IAI.73.3.1744-1753.2005 15731075 PMC1064912

[B24] Eum S.-Y. Kong J.-H. Hong M.-S. Lee Y.-J. Kim J.-H. Hwang S.-H.. (2010). Neutrophils Are the Predominant Infected Phagocytic Cells in the Airways of Patients With Active Pulmonary TB. Chest 137, 122–128. doi: 10.1378/chest.09-0903 19749004 PMC2803122

[B25] Farzeen T. (2016). Role of Low Density Neutrophils in Human Health and Disease. Trends Cell Mol. Biol. 11, 1–17.

[B26] Ferrante A. Thong Y. H. (1980). Optimal Conditions for Simultaneous Purification of Mononuclear and Polymorphonuclear Leucocytes From Human Blood by the Hypaque-Ficoll Method. J. Immunol. Methods 36, 109–117. doi: 10.1016/0022-1759(80)90036-8 7430646

[B27] Filep J. G. Ariel A. (2020). Neutrophil Heterogeneity and Fate in Inflamed Tissues: Implications for the Resolution of Inflammation. Am. J. Physiol. Cell Physiol. 319, C510–C532. doi: 10.1152/ajpcell.00181.2020 32667864 PMC7509268

[B28] Flanagan P. Lionetti F. (1955). Lysozyme Distribution in Blood. Blood 10, 497–501. doi: 10.1182/blood.V10.5.497.497 14363330

[B29] Flynn J. L. Goldstein M. M. Chan J. Triebold K. J. Pfeffer K. Lowenstein C. J.. (1995). Tumor Necrosis Factor-Alpha is Required in the Protective Immune Response Against Mycobacterium Tuberculosis in Mice. Immunity 2, 561–572. doi: 10.1016/1074-7613(95)90001-2 7540941

[B30] Fortunati E. Kazemier K. M. Grutters J. C. Koenderman L. Van den Bosch V. J. M. M. (2009). Human Neutrophils Switch to an Activated Phenotype After Homing to the Lung Irrespective of Inflammatory Disease. Clin. Exp. Immunol. 155, 559–566. doi: 10.1111/j.1365-2249.2008.03791.x 19077082 PMC2669533

[B31] Fridlender Z. G. Sun J. Kim S. Kapoor V. Cheng G. Ling L.. (2009). Polarization of Tumor-Associated Neutrophil (TAN) Phenotype by TGF-β: “N1” Versus “N2” TAN. Cancer Cell 16, 183–194. doi: 10.1016/j.ccr.2009.06.017 19732719 PMC2754404

[B32] Gideon H. P. Phuah J. Junecko B. A. Mattila J. T. (2019). Neutrophils Express Pro- and Anti-Inflammatory Cytokines in Granulomas From Mycobacterium Tuberculosis-Infected Cynomolgus Macaques. Mucosal Immunol. 12, 1370–1381. doi: 10.1038/s41385-019-0195-8 31434990 PMC6824993

[B33] Gómez-Reino J. J. Carmona L. Valverde V. R. Mola E. M. Montero M. D. BIOBADASER Group (2003). Treatment of Rheumatoid Arthritis With Tumor Necrosis Factor Inhibitors may Predispose to Significant Increase in Tuberculosis Risk: A Multicenter Active-Surveillance Report. Arthritis Rheum. 48, 2122–2127. doi: 10.1002/art.11137 12905464

[B34] Gopal R. Monin L. Torres D. Slight S. Mehra S. McKenna K. C.. (2013). S100A8/A9 Proteins Mediate Neutrophilic Inflammation and Lung Pathology During Tuberculosis. Am. J. Respir. Crit. Care Med. 188, 1137–1146. doi: 10.1164/rccm.201304-0803OC 24047412 PMC3863739

[B35] Grieshaber-Bouyer R. Nigrovic P. A. (2019). Neutrophil Heterogeneity as Therapeutic Opportunity in Immune-Mediated Disease. Front. Immunol. 10. doi: 10.3389/fimmu.2019.00346 PMC6409342 30886615

[B36] Hacbarth E. Kajdacsy-Balla A. (1986). Low Density Neutrophils in Patients With Systemic Lupus Erythematosus, Rheumatoid Arthritis, and Acute Rheumatic Fever. Arthritis Rheumatism 29, 1334–1342. doi: 10.1002/art.1780291105 2430586

[B37] Hardisty G. R. Llanwarne F. Minns D. Gillan J. L. Davidson D. J. Gwyer Findlay E.. (2021). High Purity Isolation of Low Density Neutrophils Casts Doubt on Their Exceptionality in Health and Disease. Front. Immunol. 12. doi: 10.3389/fimmu.2021.625922 PMC8217868 34168640

[B38] Hartl D. Krauss-Etschmann S. Koller B. Hordijk P. L. Kuijpers T. W. Hoffmann F.. (2008). Infiltrated Neutrophils Acquire Novel Chemokine Receptor Expression and Chemokine Responsiveness in Chronic Inflammatory Lung Diseases. J. Immunol. 181, 8053–8067. doi: 10.4049/jimmunol.181.11.8053 19017998

[B39] Hashemian S. M. R. Mortaz E. Tabarsi P. Jamaati H. Maghsoomi Z. Khosravi A.. (2014). Elevated CXCL-8 Expression in Bronchoalveolar Lavage Correlates With Disease Severity in Patients With Acute Respiratory Distress Syndrome Resulting From Tuberculosis. J. Inflamm. 11, 21. doi: 10.1186/1476-9255-11-21 PMC4126912 25110464

[B40] Hassani M. Hellebrekers P. Chen N. van Aalst C. Bongers S. Hietbrink F.. (2020). On the Origin of Low-Density Neutrophils. J. Leukoc. Biol. 107, 809–818. doi: 10.1002/JLB.5HR0120-459R 32170882 PMC7318192

[B41] Hu N. Westra J. Huitema M. G. Bijl M. Brouwer E. Stegeman C. A.. (2009). Coexpression of CD177 and Membrane Proteinase 3 on Neutrophils in Antineutrophil Cytoplasmic Autoantibody–Associated Systemic Vasculitis: Anti–proteinase 3–Mediated Neutrophil Activation Is Independent of the Role of CD177-Expressing Neutrophils. Arthritis Rheumatism 60, 1548–1557. doi: 10.1002/art.24442 19404956

[B42] Huynh J. P. Lin C.-C. Kimmey J. M. Jarjour N. N. Schwarzkopf E. A. Bradstreet T. R.. (2018). Bhlhe40 is an Essential Repressor of IL-10 During Mycobacterium Tuberculosis Infection. J. Exp. Med. 215, 1823–1838. doi: 10.1084/jem.20171704 29773644 PMC6028511

[B43] Jamil B. Shahid F. Hasan Z. Nasir N. Razzaki T. Dawood G.. (2007). Interferon Gamma/IL10 Ratio Defines the Disease Severity in Pulmonary and Extra Pulmonary Tuberculosis. Tuberculosis (Edinb) 87, 279–287. doi: 10.1016/j.tube.2007.03.004 17532265

[B44] Jones T. P. W. Dabbaj S. Mandal I. Cleverley J. Cash C. Lipman M. C. I.. (2021). The Blood Neutrophil Count After 1 Month of Treatment Predicts the Radiologic Severity of Lung Disease at Treatment End. Chest 160, 2030–2041. doi: 10.1016/j.chest.2021.07.041 34331904

[B45] Keeter W. C. Moriarty A. Mehrpouya-Baharami P. Melo P. Nadler J. Serezani C. H.. (2020). STAT4 Promotes Critical Neutrophil Functions and is Required for Antimicrobial Immunity in Mice. J. Immunol. 204, 148.22–148.22.

[B46] Keller C. Hoffmann R. Lang R. Brandau S. Hermann C. Ehlers S. (2006). Genetically Determined Susceptibility to Tuberculosis in Mice Causally Involves Accelerated and Enhanced Recruitment of Granulocytes. Infect. Immun. 74, 4295–4309. doi: 10.1128/IAI.00057-06 16790804 PMC1489748

[B47] Kimmey J. M. Huynh J. P. Weiss L. A. Park S. Kambal A. Debnath J.. (2015). Unique Role for ATG5 in Neutrophil-Mediated Immunopathology During M. Tuberculosis Infection. Nature 528, 565–569. doi: 10.1038/nature16451 26649827 PMC4842313

[B48] Kishimoto T. K. Jutila M. A. Berg E. L. Butcher E. C. (1989). Neutrophil Mac-1 and MEL-14 Adhesion Proteins Inversely Regulated by Chemotactic Factors. Science 245, 1238–1241. doi: 10.1126/science.2551036 2551036

[B49] Krupa A. Fol M. Dziadek B. R. Kepka E. Wojciechowska D. Brzostek A.. (2015). Binding of CXCL8/IL-8 to Mycobacterium Tuberculosis Modulates the Innate Immune Response. Mediators Inflamm. 2015, 124762. doi: 10.1155/2015/124762 26300588 PMC4537748

[B50] La Manna M. P. Orlando V. Paraboschi E. M. Tamburini B. Di Carlo P. Cascio A.. (2019). Mycobacterium Tuberculosis Drives Expansion of Low-Density Neutrophils Equipped With Regulatory Activities. Front. Immunol. 10. doi: 10.3389/fimmu.2019.02761 PMC6892966 31849955

[B51] Larsen C. G. Thomsen M. K. Gesser B. Thomsen P. D. Nowak J. Skødt V.. (1995). The Delayed-Type Hypersensitivity Reaction is Dependent on IL-8. Inhibition tuberculin skin reaction by an anti-IL-8 monoclonal antibody. 8, 2151–7. 7636263

[B52] Lázár-Molnár E. Chen B. Sweeney K. A. Wang E. J. Liu W. Lin J.. (2010). Programmed Death-1 (PD-1)–Deficient Mice are Extraordinarily Sensitive to Tuberculosis. PNAS 107, 13402–13407. doi: 10.1073/pnas.1007394107 20624978 PMC2922129

[B53] Lee J.-S. Kim K. H. Lee D.-Y. Choi H.-H. Lee H.-M. Son J. W.. (2008). Depressed CCL5 Expression in Human Pulmonary Tuberculosis. J. Bacteriol Virol. 38, 97. doi: 10.4167/jbv.2008.38.3.97

[B54] Li Y. Li H. Wang H. Pan H. Zhao H. Jin H.. (2019). The Proportion, Origin and Pro-Inflammation Roles of Low Density Neutrophils in SFTS Disease. BMC Infect. Dis. 19, 109. doi: 10.1186/s12879-019-3701-4 30717709 PMC6360754

[B55] Liu W. Yan M. Liu Y. McLeish K. R. Coleman W. G. Rodgers G. P. (2012). Olfactomedin 4 Inhibits Cathepsin C-Mediated Protease Activities, Thereby Modulating Neutrophil Killing of Staphylococcus Aureus and Escherichia Coli in Mice. J. Immunol. 189, 2460–2467. doi: 10.4049/jimmunol.1103179 22844115 PMC3424379

[B56] Liu W. Yan M. Sugui J. A. Li H. Xu C. Joo J.. (2013). Olfm4 Deletion Enhances Defense Against Staphylococcus Aureus in Chronic Granulomatous Disease. J. Clin. Invest. 123, 3751–3755. doi: 10.1172/JCI68453 23908114 PMC3754258

[B57] Lood C. Blanco L. P. Purmalek M. M. Carmona-Rivera C. De Ravin S. S. Smith C. K.. (2016). Neutrophil Extracellular Traps Enriched in Oxidized Mitochondrial DNA are Interferogenic and Contribute to Lupus-Like Disease. Nat. Med. 22, 146–153. doi: 10.1038/nm.4027 26779811 PMC4742415

[B58] Lovewell R. R. Baer C. E. Mishra B. B. Smith C. M. Sassetti C. M. (2021). Granulocytes Act as a Niche for Mycobacterium Tuberculosis Growth. Mucosal Immunol. 14, 229–241. doi: 10.1038/s41385-020-0300-z 32483198 PMC7704924

[B59] Lowe D. M. Demaret J. Bangani N. Nakiwala J. K. Goliath R. Wilkinson K. A.. (2018). Differential Effect of Viable Versus Necrotic Neutrophils on Mycobacterium Tuberculosis Growth and Cytokine Induction in Whole Blood. Front. Immunol. 9. doi: 10.3389/fimmu.2018.00903 PMC5934482 29755473

[B60] Marini O. Costa S. Bevilacqua D. Calzetti F. Tamassia N. Spina C.. (2017). Mature CD10+ and Immature CD10– Neutrophils Present in G-CSF–treated Donors Display Opposite Effects on T Cells. Blood 129, 1343–1356. doi: 10.1182/blood-2016-04-713206 28053192

[B61] Martineau A. R. Newton S. M. Wilkinson K. A. Kampmann B. Hall B. M. Nawroly N.. (2007). Neutrophil-Mediated Innate Immune Resistance to Mycobacteria. J. Clin. Invest. 117, 1988–1994. doi: 10.1172/JCI31097 17607367 PMC1904316

[B62] Mattila J. T. Maiello P. Sun T. Via L. E. Flynn J. L. (2015). Granzyme B-Expressing Neutrophils Correlate With Bacterial Load in Granulomas From Mycobacterium Tuberculosis-Infected Cynomolgus Macaques. Cell Microbiol. 17, 1085–1097. doi: 10.1111/cmi.12428 25653138 PMC4570831

[B63] McKenna K. C. Beatty K. M. Vicetti Miguel R. Bilonick R. A. (2009). Delayed Processing of Blood Increases the Frequency of Activated CD11b+ CD15+ Granulocytes Which Inhibit T Cell Function. J. Immunol. Methods 341, 68–75. doi: 10.1016/j.jim.2008.10.019 19041316

[B64] McNab F. W. Berry M. P. R. Graham C. M. Bloch S. A. A. Oni T. Wilkinson K. A.. (2011). Programmed Death Ligand 1 Is Over-Expressed by Neutrophils in the Blood of Patients With Active Tuberculosis: Immunity to Infection. Eur. J. Immunol. 41, 1941–1947. doi: 10.1002/eji.201141421 21509782 PMC3179592

[B65] Mehrpouya-Bahrami P. Moriarty A. K. De Melo P. Keeter W. C. Alakhras N. S. Nelson A. S.. (2021). STAT4 Is Expressed in Neutrophils and Promotes Antimicrobial Immunity. JCI Insight 6, 141326. doi: 10.1172/jci.insight.141326 34138758 PMC8410094

[B66] Midgley A. Beresford M. W. (2016). Increased Expression of Low Density Granulocytes in Juvenile-Onset Systemic Lupus Erythematosus Patients Correlates With Disease Activity. Lupus 25, 407–411. doi: 10.1177/0961203315608959 26453665

[B67] Mishra B. B. Lovewell R. R. Olive A. J. Zhang G. Wang W. Eugenin E.. (2017). Nitric Oxide Prevents a Pathogen-Permissive Granulocytic Inflammation During Tuberculosis. Nat. Microbiol. 2, 17072. doi: 10.1038/nmicrobiol.2017.72 28504669 PMC5461879

[B68] Moreira-Teixeira L. Stimpson P. J. Stavropoulos E. Hadebe S. Chakravarty P. Ioannou M.. (2020). Type I IFN Exacerbates Disease in Tuberculosis-Susceptible Mice by Inducing Neutrophil-Mediated Lung Inflammation and NETosis. Nat. Commun. 11, 5566. doi: 10.1038/s41467-020-19412-6 33149141 PMC7643080

[B69] Nair S. Huynh J. P. Lampropoulou V. Loginicheva E. Esaulova E. Gounder A. P.. (2018). Irg1 Expression in Myeloid Cells Prevents Immunopathology During M. Tuberculosis Infection. J. Exp. Med. 215, 1035–1045. doi: 10.1084/jem.20180118 29511063 PMC5881474

[B70] Nakayama F. Nishihara S. Iwasaki H. Kudo T. Okubo R. Kaneko M.. (2001). CD15 Expression in Mature Granulocytes Is Determined by α1,3-Fucosyltransferase IX, But in Promyelocytes and Monocytes by α1,3-Fucosyltransferase IV. J. Biol. Chem. 276, 16100–16106. doi: 10.1074/jbc.M007272200 11278338

[B71] Nandi B. Behar S. M. (2011). Regulation of Neutrophils by Interferon-γ Limits Lung Inflammation During Tuberculosis Infection. J. Exp. Med. 208, 2251–2262. doi: 10.1084/jem.20110919 21967766 PMC3201199

[B72] Negorev D. Beier U. H. Zhang T. Quatromoni J. G. Bhojnagarwala P. Albelda S. M.. (2018). Human Neutrophils can Mimic Myeloid-Derived Suppressor Cells (PMN-MDSC) and Suppress Microbead or Lectin-Induced T Cell Proliferation Through Artefactual Mechanisms. Sci. Rep. 8, 3135. doi: 10.1038/s41598-018-21450-6 29453429 PMC5816646

[B73] Niazi M. K. K. Dhulekar N. Schmidt D. Major S. Cooper R. Abeijon C.. (2015). Lung Necrosis and Neutrophils Reflect Common Pathways of Susceptibility to Mycobacterium Tuberculosis in Genetically Diverse, Immune-Competent Mice. Dis. Model. Mech. 8, 1141–1153. doi: 10.1242/dmm.020867 26204894 PMC4582107

[B74] Obregón-Henao A. Henao-Tamayo M. Orme I. M. Ordway D. J. (2013). Gr1(int)CD11b+ Myeloid-Derived Suppressor Cells in Mycobacterium Tuberculosis Infection. PLoS One 8, e80669. doi: 10.1371/journal.pone.0080669 24224058 PMC3815237

[B75] O’Kane C. M. Boyle J. J. Horncastle D. E. Elkington P. T. Friedland J. S. (2007). Monocyte-Dependent Fibroblast CXCL8 Secretion Occurs in Tuberculosis and Limits Survival of Mycobacteria Within Macrophages. J. Immunol. 178, 3767–3776. doi: 10.4049/jimmunol.178.6.3767 17339475

[B76] Pan Z. Z. Parkyn L. Ray A. Ray P. (2000). Inducible Lung-Specific Expression of RANTES: Preferential Recruitment of Neutrophils. Am. J. Physiol. Lung Cell Mol. Physiol. 279, L658–L666. doi: 10.1152/ajplung.2000.279.4.L658 11000125

[B77] Pedrosa J. Saunders B. M. Appelberg R. Orme I. M. Silva M. T. Cooper A. M. (2000). Neutrophils Play a Protective Nonphagocytic Role in Systemic Mycobacterium Tuberculosis Infection of Mice. Infect. Immun. 68, 577–583. doi: 10.1128/IAI.68.2.577-583.2000 10639420 PMC97179

[B78] Rao J. Su R. Peng Y. Guo Y. Huang Z. Ye Y.. (2021). Low-Density Granulocytes Affect T-SPOT.TB Assay by Inhibiting the Production of Interferon-γ in T Cells *via* PD-L1/PD-1 Pathway. Front. Microbiol. 11. doi: 10.3389/fmicb.2020.622389 PMC7876290 33584591

[B79] Rarok A. A. Stegeman C. A. Limburg P. C. Kallenberg C. G. M. (2002). Neutrophil Membrane Expression of Proteinase 3 (PR3) Is Related to Relapse in PR3-ANCA-Associated Vasculitis. JASN 13, 2232–2238. doi: 10.1097/01.ASN.0000028642.26222.00 12191967

[B80] Ravimohan S. Kornfeld H. Weissman D. Bisson G. P. (2018). Tuberculosis and Lung Damage: From Epidemiology to Pathophysiology. Eur. Respir. Rev. 27, 170077. doi: 10.1183/16000617.0077-2017 29491034 PMC6019552

[B81] Reyes L. Sanchez-Garcia A. Morrison T. Howden A. J. M. Watts E. R. Arienti S.. (2021). A Type I IFN, Prothrombotic Hyperinflammatory Neutrophil Signature is Distinct for COVID-19 ARDS. Wellcome Open Res. 6, 38. doi: 10.12688/wellcomeopenres.16584.2 33997298 PMC8112464

[B82] Rieber N. Wecker I. Neri D. Fuchs K. Schäfer I. Brand A.. (2014). Extracorporeal Photopheresis Increases Neutrophilic Myeloid-Derived Suppressor Cells in Patients With GvHD. Bone Marrow Transplant. 49, 545–552. doi: 10.1038/bmt.2013.236 24464140

[B83] Scapini P. Marini O. Tecchio C. Cassatella M. A. (2016). Human Neutrophils in the Saga of Cellular Heterogeneity: Insights and Open Questions. Immunol. Rev. 273, 48–60. doi: 10.1111/imr.12448 27558327

[B84] Schmidt T. Zündorf J. Grüger T. Brandenburg K. Reiners A.-L. Zinserling J.. (2012). CD66b Overexpression and Homotypic Aggregation of Human Peripheral Blood Neutrophils After Activation by a Gram-Positive Stimulus. J. Leukocyte Biol. 91, 791–802. doi: 10.1189/jlb.0911483 22319104

[B85] Schmielau J. Finn O. J. (2001). Activated Granulocytes and Granulocyte-Derived Hydrogen Peroxide are the Underlying Mechanism of Suppression of T-Cell Function in Advanced Cancer Patients. Cancer Res. 61, 4756–4760. 11406548

[B86] Scott N. R. Swanson R. V. Al-Hammadi N. Domingo-Gonzalez R. Rangel-Moreno J. Kriel B. A.. (2020). S100A8/A9 Regulates CD11b Expression and Neutrophil Recruitment During Chronic Tuberculosis. J. Clin. Invest. 130, 3098–3112. doi: 10.1172/JCI130546 32134742 PMC7259997

[B87] Seman B. G. Robinson C. M. (2021). The Enigma of Low-Density Granulocytes in Humans: Complexities in the Characterization and Function of LDGs During Disease. Pathogens 10, 1091. doi: 10.3390/pathogens10091091 34578124 PMC8470838

[B88] Seman B. G. Vance J. K. Akers S. M. Robinson C. M. (2021). Neonatal Low-Density Granulocytes Internalize and Kill Bacteria But Suppress Monocyte Function Using Extracellular DNA. J. Cell Sci. 134, jcs252528. doi: 10.1242/jcs.252528 33589502

[B89] Silvestre-Roig C. Fridlender Z. G. Glogauer M. Scapini P. (2019). Neutrophil Diversity in Health and Disease. Trends Immunol. 40, 565–583. doi: 10.1016/j.it.2019.04.012 31160207 PMC7185435

[B90] Stegelmann F. Bastian M. Swoboda K. Bhat R. Kiessler V. Krensky A. M.. (2005). Coordinate Expression of CC Chemokine Ligand 5, Granulysin, and Perforin in CD8 ^+^ T Cells Provides a Host Defense Mechanism Against *Mycobacterium Tuberculosis* . J. Immunol. 175, 7474–7483. doi: 10.4049/jimmunol.175.11.7474 16301655

[B91] Subbian S. Bandyopadhyay N. Tsenova L. O’Brien P. Khetani V. Kushner N. L.. (2013). Early Innate Immunity Determines Outcome of Mycobacterium Tuberculosis Pulmonary Infection in Rabbits. Cell Commun. Signal 11, 60. doi: 10.1186/1478-811X-11-60 23958185 PMC3765177

[B92] Sugawara I. Yamada H. Li C. Mizuno S. Takeuchi O. Akira S. (2003). Mycobacterial Infection in TLR2 and TLR6 Knockout Mice. Microbiol. Immunol. 47, 327–336. doi: 10.1111/j.1348-0421.2003.tb03404.x 12825894

[B93] Sugawara I. Yamada H. Mizuno S. (2004). STAT1 Knockout Mice Are Highly Susceptible to Pulmonary Mycobacterial Infection. Tohoku J. Exp. Med. 202, 41–50. doi: 10.1620/tjem.202.41 14738323

[B94] Sun R. (2022). Dysfunction of Low-Density Neutrophils in Peripheral Circulation in Patients With Sepsis. Sci. Rep. 10, 685. doi: 10.1038/s41598-021-04682-x PMC8758723 35027618

[B95] Su R. Peng Y. Deng Z. Deng Y. Ye J. Guo Y.. (2019). Mycobacterium Tuberculosis Infection Induces Low-Density Granulocyte Generation by Promoting Neutrophil Extracellular Trap Formation *via* ROS Pathway. Front. Microbiol. 10 1468. doi: 10.3389/fmicb.2019.01468 PMC6637951 31354639

[B96] Takizawa S. Murao A. Ochani M. Aziz M. Wang P. (2021). Frontline Science: Extracellular CIRP Generates a Proinflammatory Ly6G+ CD11bhi Subset of Low-Density Neutrophils in Sepsis. J. Leukoc. Biol. 109, 1019–1032. doi: 10.1002/JLB.3HI0620-416R 33070370 PMC8053202

[B97] Tsiganov E. N. Verbina E. M. Radaeva T. V. Sosunov V. V. Kosmiadi G. A. Nikitina I. Y.. (2014). Gr-1dimcd11b+ Immature Myeloid-Derived Suppressor Cells But Not Neutrophils are Markers of Lethal Tuberculosis Infection in Mice. J. Immunol. 192, 4718–4727. doi: 10.4049/jimmunol.1301365 24711621 PMC4537794

[B98] Valadez-Cosmes P. Maitz K. Kindler O. Raftopoulou S. Kienzl M. Santiso A.. (2021). Identification of Novel Low-Density Neutrophil Markers Through Unbiased High-Dimensional Flow Cytometry Screening in Non-Small Cell Lung Cancer Patients. Front. Immunol. 12. doi: 10.3389/fimmu.2021.703846 PMC8414579 34484199

[B99] van Lochem E. G. van der Velden V. H. J. Wind H. K. te Marvelde J. G. Westerdaal N. A. C. van Dongen J. (2004). Immunophenotypic Differentiation Patterns of Normal Hematopoiesis in Human Bone Marrow: Reference Patterns for Age-Related Changes and Disease-Induced Shifts. Cytometry Part B: Clin. Cytometry 60B, 1–13. doi: 10.1002/cyto.b.20008 15221864

[B100] Verbon A. Juffermans N. Van Deventer S. J. H. Speelman P. Van Deutekom H. van der Poll T. (1999). Serum Concentrations of Cytokines in Patients With Active Tuberculosis (TB) and After Treatment. Clin. Exp. Immunol. 115, 110–113. doi: 10.1046/j.1365-2249.1999.00783.x 9933428 PMC1905191

[B101] Vesosky B. Rottinghaus E. K. Stromberg P. Turner J. Beamer G. (2010). CCL5 Participates in Early Protection Against Mycobacterium Tuberculosis. J. Leukoc. Biol. 87, 1153–1165. doi: 10.1189/jlb.1109742 20371596 PMC2872537

[B102] Zhang G. deWeerd N. A. Stifter S. A. Liu L. Zhou B. Wang W.. (2018). A Proline Deletion in IFNAR1 Impairs IFN-Signaling and Underlies Increased Resistance to Tuberculosis in Humans. Nat. Commun. 9, 85. doi: 10.1038/s41467-017-02611-z 29311663 PMC5758831

